# Access to innovative therapies in pediatric oncology: Report of the nationwide experience in Canada

**DOI:** 10.1002/cam4.7033

**Published:** 2024-02-24

**Authors:** Sandra Judd, Gabriel Revon‐Riviere, Stephanie A. Grover, Rebecca J. Deyell, Magimairajan Issai Vanan, Victor A. Lewis, Lucie Pecheux, Alexandra P. Zorzi, Catherine Goudie, Raoul Santiago, Thai Hoa Tran, Lesleigh S. Abbott, Josee Brossard, Paul Moorehead, Saima Alvi, Carol Portwine, Avram Denburg, James A. Whitlock, Sarah Cohen‐Gogo, Daniel A. Morgenstern

**Affiliations:** ^1^ Department of Pharmacy Hospital for Sick Children Toronto Ontario Canada; ^2^ Division of Haematology/Oncology, Hospital for Sick Children, Department of Pediatrics University of Toronto Toronto Ontario Canada; ^3^ Genetics and Genome Biology, Hospital for Sick Children Toronto Ontario Canada; ^4^ Division of Pediatric Hematology Oncology BMT BC Children's Hospital and Research Institute Vancouver British Columbia Canada; ^5^ Pediatric Neuro‐Oncology, Division of Pediatric Hematology‐Oncology, Cancer Care Manitoba University of Manitoba Winnipeg Manitoba Canada; ^6^ University of Calgary Alberta Canada; ^7^ Stollery Children's Hospital University of Alberta Edmonton Alberta Canada; ^8^ Department of Pediatrics, Children's Hospital London Health Sciences Centre Western University London Ontario Canada; ^9^ Department of Pediatrics, Division of Hematology‐Oncology, Montreal Children's Hospital McGill University Health Centre Québec Canada; ^10^ Department of Pediatrics, CHU de Québec Laval University Québec Canada; ^11^ Division of Pediatric Hematology‐Oncology Charles‐Bruneau Cancer Center, CHU Sainte‐Justine Montreal Québec Canada; ^12^ Division of Hematology/Oncology Children's Hospital of Eastern Ontario Ottawa Ontario Canada; ^13^ Department of Pediatrics CHU de Sherbrooke, Univesité de Sherbrooke Sherbrooke Québec Canada; ^14^ Department of Pediatrics, Janeway Children's Health and Rehabilitation Centre Memorial University of Newfoundland St. John's Newfoundland and Labrador Canada; ^15^ Pediatric Hematology/Oncology, Jim Pattison Children's Hospital Saskatoon Saskatchewan Canada; ^16^ McMaster Children's Hospital McMaster University Hamilton Ontario Canada

**Keywords:** immunotherapy, precision oncology, real‐world data, targeted therapy

## Abstract

**Background:**

The need for new therapies to improve survival and outcomes in pediatric oncology along with the lack of approval and accessible clinical trials has led to “out‐of‐trial” use of innovative therapies. We conducted a retrospective analysis of requests for innovative anticancer therapy in Canadian pediatric oncology tertiary centers for patients less than 30 years old between 2013 and 2020.

**Methods:**

Innovative therapies were defined as cancer‐directed drugs used (a) off‐label, (b) unlicensed drugs being used outside the context of a clinical trial, or (c) approved drugs with limited evidence in pediatrics. We excluded cytotoxic chemotherapy, cellular products, and cytokines.

**Results:**

We retrieved data on 352 innovative therapy drug requests. Underlying diagnosis was primary CNS tumor 31%; extracranial solid tumor 37%, leukemia/lymphoma 22%, LCH 2%, and plexiform neurofibroma 6%. RAS/MAP kinase pathway inhibitors were the most frequently requested innovative therapies in 28% of all requests followed by multi‐targeted tyrosine kinase inhibitors (17%), inhibitors of the PIK3CA‐mTOR‐AKT pathway (8%), immune checkpoints inhibitors (8%), and antibody drug conjugates (8%). In 112 out of 352 requests, innovative therapies were used in combination with another anticancer agent. 48% of requests were motivated by the presence of an actionable molecular target. Compassionate access accounted for 52% of all requests while public insurance was used in 27%. Mechanisms of funding varied between provinces.

**Conclusion:**

This real‐world data collection illustrates an increasing use of “out‐of‐trial” innovative therapies in pediatric oncology. This new field of practice warrants further studies to understand the impact on patient trajectory and equity in access to innovative therapies.

## 1 INTRODUCTION

The need for new therapies to improve survival and outcomes of children and adolescents with cancer is well recognized but insufficiently addressed.^1^, ^2^, ^3^ With a few notable exceptions (e.g., anti‐GD2 antibody therapy for neuroblastoma^4^), new therapies for childhood cancers typically arise from adult oncology‐oriented research. Pediatric clinical trials and ultimately approval for pediatric‐specific indications often lag years behind adult development, resulting in significant challenges in accessing new treatment options for children and adolescents.^5^

In many countries, including Canada and much of Europe, marketing authorization from regulatory authorities (e.g., Health Canada or European Medicines Agency) is insufficient for a given therapy to become routinely available. A health technology assessment (HTA) incorporating a health economics evaluation is necessary before reimbursement from the public‐payer health system.^6^ The current processes of regulatory and HTA approval, which are routinely premised on adult indications, often constrain licensing and public reimbursement for pediatric‐specific indications, even where evidence of efficacy in the pediatric setting exists. For example, in 2020 when this study was initiated, dabrafenib was approved in Canada for adult patients with melanoma and non‐small cell lung cancer but not approved for patients with *BRAF* V600E‐mutated gliomas or Langerhans cell histiocytosis (LCH), despite demonstrated improvement of patients' outcomes.^7^ Regorafenib is approved for adult patients with colorectal cancer, gastrointestinal stromal tumors, and hepatocellular carcinoma while data are also available regarding potential clinical benefit in patients, including pediatric, with bone sarcomas.^8^ The level of evidence supporting the use of these compounds in pediatrics is lower than the evidence supporting their adult indication. However, despite small numbers and early phase methodology, these therapies are considered superior to the existing standard of care resulting in strong consensus for clinical use. Progress in precision oncology has led to the identification of numerous novel potential therapeutic targets.^9^ This approach has led to major successes in treatment and commercialization of new drugs^10^ but also raised unprecedented challenges in the generation of scientific evidence to support the use of and access to these innovative therapies.

In Canada, similar to other high‐income countries, many potential routes are available to access innovative therapies outside of clinical trials (“out of trial”). Innovative therapies may be prescribed as off‐label medications, when approved and marketed for another indication, although in these circumstances the drug costs are unlikely to be covered by public or private health insurance plans. When not approved or marketed in Canada, innovative therapies marketed for use in other countries in the context of serious, life‐threatening conditions may be accessed through Health Canada's Special Access Program often with a pharmaceutical company‐managed compassionate access program. Pediatric‐friendly dosage forms (e.g., oral liquids, dispersible tablets) still under investigation may be made available for pediatric patients through a similar mechanism. When accessed at an early stage of development, with limited safety data in the pediatric population, or in the context of novel combinations, innovative therapies can be accessed through the single patient study mechanism.^11^

The pediatric oncology community have consistently stated that the preferred route of access to innovative therapies is participation in a clinical trial.^12^ However, recognition is growing that this is not always feasible and that there are frequent uses of innovative therapies “out of trial” in pediatric oncology. Previous studies have described the indications, safety, and efficacy of innovative therapies accessed through off‐label^13^ or compassionate use^14^, ^15^ based on retrospective studies in large pediatric oncology centers. In this work, we provide pan‐Canadian real‐world data on the use of innovative therapies for pediatric oncology patients “out of trial”. Our primary aim was to describe which innovative therapies were accessed, together with their matching molecular targets when relevant. We also studied routes of access, application processes, approval timeframe, and funding mechanisms.

## 2 METHODS

### 2.1 Case selection

We conducted a retrospective analysis of innovative anticancer therapy drug access requests for patients aged 0–29 years in 2013–2020 (date of Research Ethics Board approval as a retrospective study) from Canadian pediatric oncology tertiary care centers. Innovative therapies were defined as cancer‐directed drugs used off‐label, unlicensed drugs being used “out of trial”, or Health Canada approved drugs with limited clinical experience, evidence of safety in pediatrics, or indication for use at the time of the request. Drug access requests for non‐cancer‐directed therapy, supportive care therapy or a therapy for a comorbid condition were excluded. In this analysis, we excluded requests for cytotoxic chemotherapy, cellular products, and cytokines to focus on targeted therapies, antibody drug conjugates (ADCs) and other immunotherapy products.

### 2.2 Data collection

Data were abstracted from electronic and paper charts, pharmacy records, provincial funding databases, and interviews with oncologists. Study data were collected using REDCap electronic data capture tools^16^, ^17^ hosted at The Hospital for Sick Children, Toronto, Ontario, Canada. We collected the reason for innovative therapies request, patient characteristics and disease, medical indication, approval timelines, and potential impact on clinical status. Presence of molecular targets and rationale were captured as determined by the treating/reporting clinician. Respondents completed one survey for each drug access request. If a combination of innovative therapies was requested for a patient, respondents submitted separate responses for each therapy.

### 2.3 Ethics statement

Ethics approval and a waiver of the requirement for informed consent was received from the Hospital for Sick Children Research Ethics Board (REB) and REBs at participating centers.

## 3 RESULTS

**FIGURE 1:**
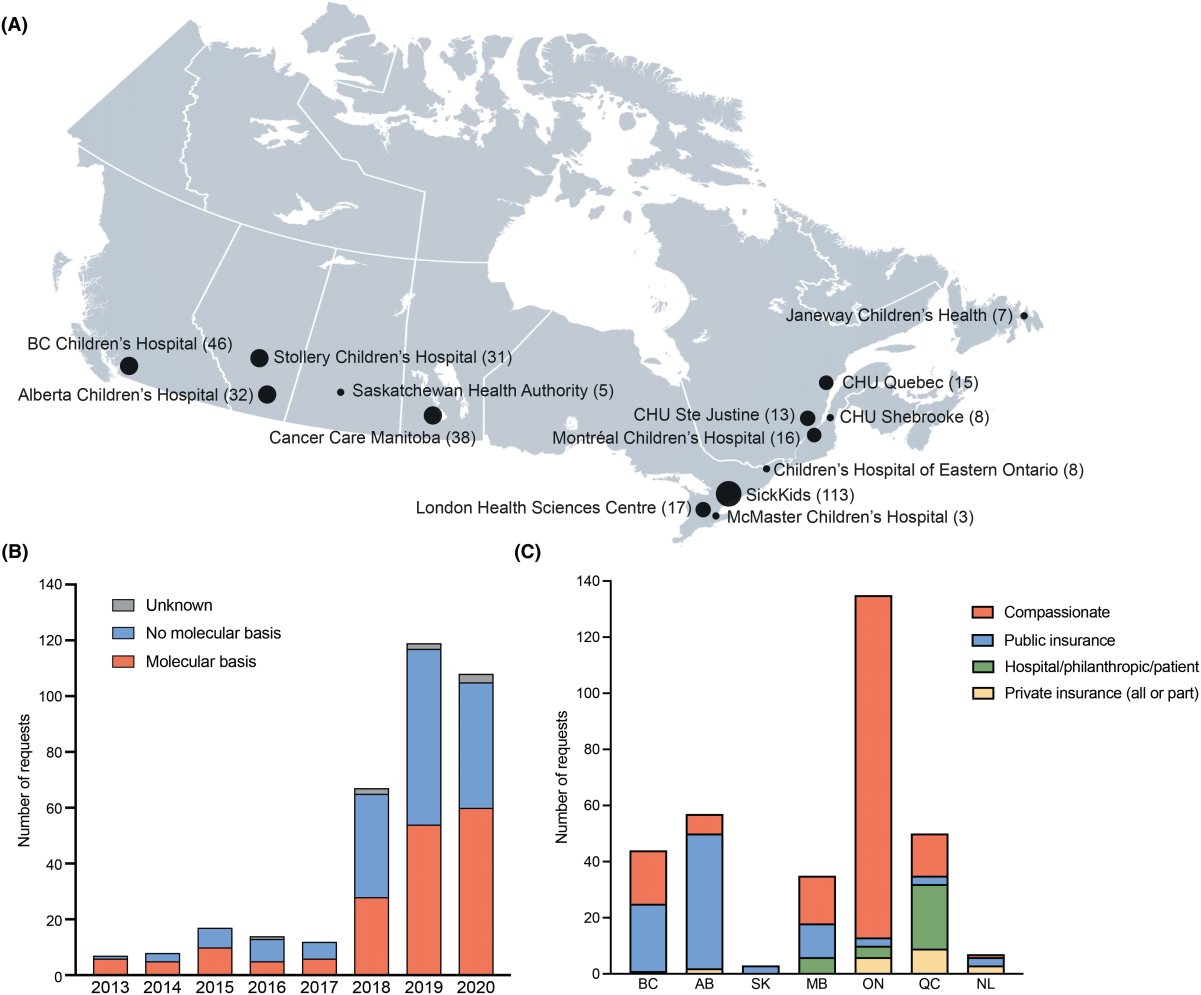
(A) Geographic representation of the number of centers participating in the study and number of requests per site. Provinces from west to east: British Colombia (BC), 1 site; Alberta (AB), 2 sites; Saskatchewan (SK), 1 site; Manitoba (MB), 1 site; Ontario (ON), 4 sites; Quebec (QC), 4 sites; Newfoundland (NL), 1 site. (B) Number of submitted drug access requests by year and proportion of molecularly informed requests. Data for 2020 may be incomplete due to the date of ethics approval at each site. Data prior to 2018 may have a recall bias due to implementation of electronic medical records at several institutions and provincial submission databases in recent years. (C) Routes of access by province. Includes all funding mechanisms reported for approved drug access requests (*n* = 303 of the 331 approved requests). Respondents were able to select multiple funding mechanisms utilized for each drug access request, with 338 funding mechanisms reported for 303 requests.

This study includes data on 352 innovative drug access requests representing 330 lines of treatment from 14 centers (Figure 1A). Although data were collected from 2013 onwards, 84% of the requests (294) were from 2018 to 2020 (Figure 1B). At the time of request, 49% of patients were 0–11 years old, 42% 12–17 years and 7% >18 [2% undisclosed age]. The patient's underlying diagnosis prompting the request were 31% primary CNS tumor (predominantly gliomas (88/109)), 37% extracranial solid tumor (bone sarcomas (48/130) and soft tissue sarcomas (36/130)), 22% leukemia and lymphoma (leukemia (65/78)), 2.3% LCH, and 6.5% plexiform neurofibroma [1.1% undisclosed diagnosis].

During the study period, requests for cytotoxic chemotherapy, cellular products, and cytokines were inconsistently captured and showed dramatic heterogeneity between centers. Requests reported included tisagenleucel (2), aldesleukin (11), dinutuximab (6), interferon alpha (4) and cytotoxic chemotherapy (clofarabine, treosulfan, temozolomide, pegylated liposomal doxorubicin, nelarabine, cladribine, thiotepa, azacitidine and trabectidine, 45 requests in total). We excluded these from the analysis.

### 3.1 Innovative therapies and molecular targets

**FIGURE 2:**
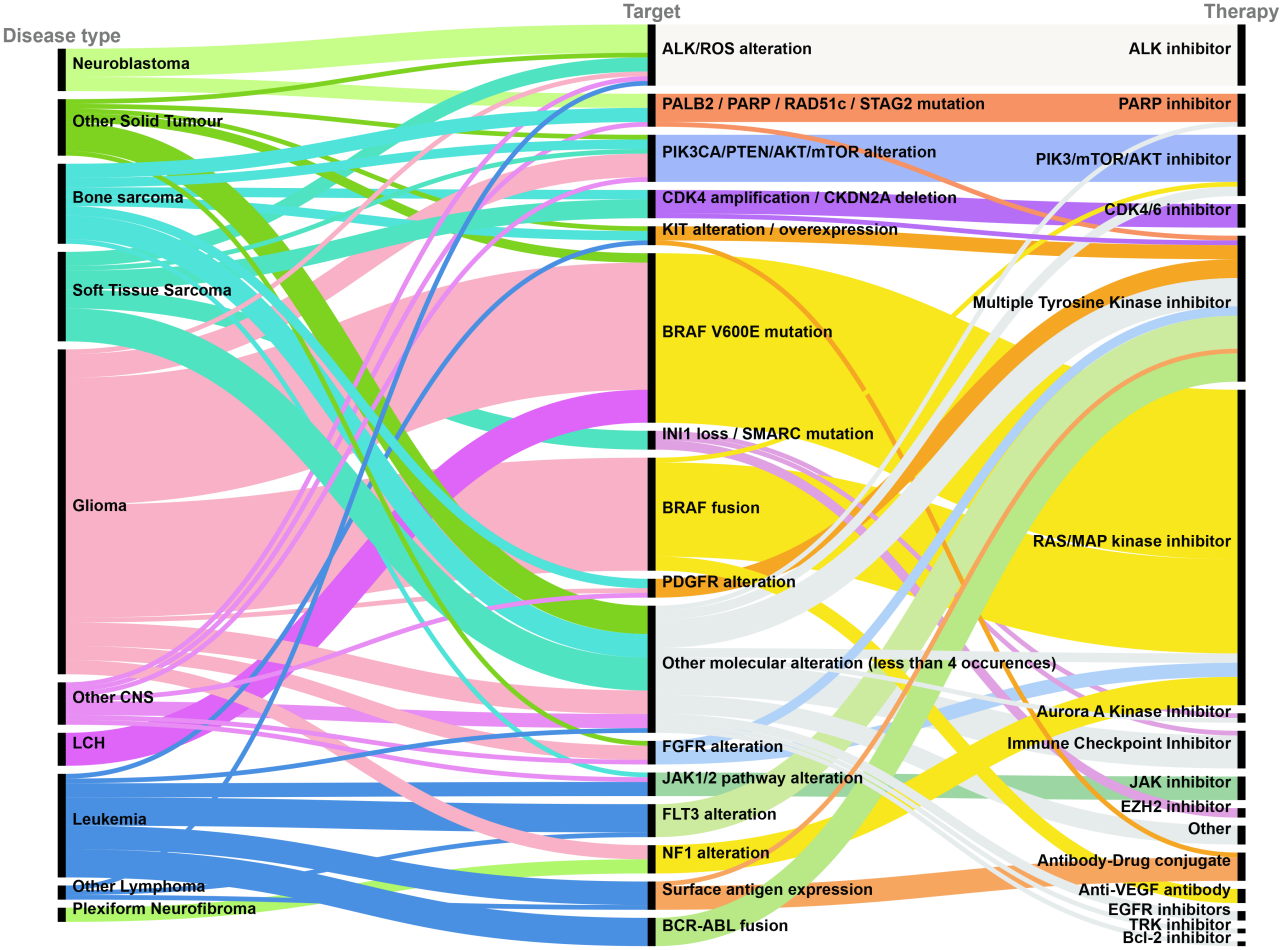
Correlation of disease type, reported molecular target, and requested innovative therapy (*n* = 170). The height of each black block is proportional to the number of requests for each disease, molecular target and innovative therapy drug class. Colors for visual separation only.

The most frequently requests were for RAS/MAP kinase pathway (28%), multi‐targeted tyrosine kinase (17%), PIK3CA‐mTOR‐AKT pathway (8%), immune checkpoint inhibitors (8%) and ADCs (8%). Drug requests by drug class as single agent and combinations are presented in Table 1. In 32% of requests (112/352), innovative therapies were used in combination with another agent. In 23 requests, this other agent was also an innovative therapy (e.g., trametinib–dabrafenib) (Table 1). In 66 requests, innovative therapy was used in combination with another anticancer treatment, including chemotherapy, steroids, or radiotherapy (including 131I‐mIBG). The presence of an actionable molecular target (as determined by the treating/reporting physician) was the motivation for 48.3% (170/352) of requests (Table 2; Figure 2). The actionable molecular targets reported included genetic point mutations, amplifications, fusions, and gene overexpression. Specific surface antigens, T‐cell score, and PDL1/CTLA4 expression were also reported as targets. Details of the techniques used to identify these molecular changes were not collected.

**TABLE 1:** Innovative therapies requests by drug class (*n*).

RAS/MAP kinase inhibitors		Multi‐targeted tyrosine kinase inhibitors		PIK3/mTOR/AKT inhibitors		Other	
Trametinib	56	Regorafenib	16	Everolimus	12	Bortezomib	2
Dabrafenib	26	Pazopanib	15	Sirolimus	9	Metformin	2
Selumetinib	12	Sorafenib	13	Temsirolimus	4	Tocilizumab	1
Vemurafenib	3	Cabozantinib	8	Alpelisib	3	Pertuzumab	1
		Axitinib	3	Ipatasertib	1	Daratumumab	1
		Sunitinib	2				
		Lenvatinib	2				
		Vandetanib	1				

Immune checkpoint inhibitors		Antibody‐drug conjugates		ALK/ROS inhibitors		BCR‐ABL inhibitors	
Nivolumab	20	Inotuzumab ozogamicin	12	Crizotinib	7	Dasatinib	12
Ipilimumab	5	Gemtuzumab ozogamicin	11	Ceritinib	5	Imatinib	3
Pembrolizumab	4	Brentuximab vedotin	5	Lorlatinib	1	Ponatinib	1

Anti‐VEGF antibody		BiTE		BCL2 inhibitors		JAK inhibitors	
Bevacizumab	14	Blinatumomab	11	Venetoclax	9	Ruxolitinib	7

CDK and cell cycle inhibitors		PARP inhibitors		EGFR inhibitors		Epigenetic regulators	
Palbociclib	7	Olaparib	6	Afatinib	1	Tazemetostat	4
Ribocilib	1	Talazoparib	1	Nimotuzumab	1	Vorinostat	1

Aurora kinase Inhibitors		FLT3 inhibitors		TRK inhibitors		Exportin inhibitors	
Alisertib	5	Midostaurin	3	Selitrectinib	1	Selinexor	1

Combinations of innovative therapies requested			
Dabrafenib–Trametinib	10	Vorinostat–Bevacizumab	1
Nivolumab–Axitinib	3	Everolimus–Bevacizumab	1
Nivolumab–Ipilimumab	4	Everolimus–Ponatinib	1
Lenvatinib–Pembrolizumab	1	Everolimus–Trametinib	1
		Everolimus–Alpelisib	1

*Note*: Each drug access request in combination is also included in the total number of requests for each drug above.

**TABLE 2:** Access to innovative therapy based on molecular information (*n* = 170).

Reported pathway alteration^a^	Number of requests	Innovative therapy requested (*n*)
BRAF V600E mutation	36	Dabrafenib (22), trametinib (13), vemurafenib (1)
BRAF fusion	24	Trametinib (19), bevacizumab (3), dabrafenib (1), everolimus (1)
ALK/ROS alteration	13	Crizotinib (7), ceritinib (5), lorlatinib (1)
PIK3CA/PTEN/AKT/mTOR alteration	10	Alpelisib (3), everolimus (3), sirolimus (3), ipatasertib (1)
FLT3 alteration	7	Sorafenib (6), midostaurin (1)
PALB2/PARP/RAD51c/STAG2 mutation	7	Olaparib (5), talazoparib (1), pazopanib (1)
*BCR*:*ABL1* fusion	6	Dasatinib (6)
CDK4 amplification, CDKN2A deletion	6	Palbociclib (5), regorafenib (1)
NF1 alteration	6	Trametinib (5), selumetinib (1)
Surface antigen expression	6	Gemtuzumab (4), brentuximab (1), imatinib (1)
FGFR alteration	5	Trametinib (3), ponatinib (1), sorafenib (1)
JAK1/2 pathway alteration	5	Ruxolitinib (5)
INI1 loss/SMARC mutation	4	Tazemetostat (2), alisertib (1), nivolumab (1)
KIT alteration/overexpression	4	Gemtuzumab (1), sunitinib (1), pazopanib (2)
PDGFR mutation	4	Dasatinib (2), regorafenib (1), pazopanib (1)
VEGF amplification/overexpression	3	Pazopanib (3)
ERC1‐BRAF fusion	2	Trametinib (2)
High T‐Cell score + PDL1/CTLA4 expression	2	Nivolumab (2)
High TMB	2	Nivolumab (2)
MSH2 and MSH6 loss	2	Ipilimumab (1), nivolumab (1)
N‐MYC amplification	2	Alisertib (1), nivolumab (1)
ABL2 and DDR2 amplification	1	Dasatinib (1)
EGFR alteration	1	Nimotuzumab (1)
ErbB2 alteration	1	Pertuzumab (1)
ErbB4 alteration	1	Afatinib (1)
*ETV6*:*NTRK3* fusion	1	Selitrectinib (1)
High HRD score	1	Olaparib (1)
NF2 alteration	1	Everolimus (1)
*RUNX1*:*ETS2* fusion	1	Venetoclax (1)
TSC2 alteration	1	Sirolimus (1)
IL‐6 overexpression	1	Tocilizumab (1)
IGF2 and SOX4 overexpression	1	Metformin (1)
IRS‐1 overexpression	1	Metformin (1)
RET alteration	1	Vandetanib (1)

*Note*: 170 out of 352 innovative drug access requests were molecularly informed.

^a^ Pathway alteration were reported here with as much detail as was reported by survey respondents.

### 3.2 Reasons to access “out‐of‐trial” innovative drugs

In 62% of requests, the reason for requesting an innovative drug “out of trial” was the lack of an accessible clinical trial for a given patient at a given time. This was noted to include a lack of relevant clinical trial options nationally, relevant clinical trial at their treatment center, or available enrollment slots in a local active trial. Various other reasons were provided for why access was being sought “out of trial”, including patient withdrawal from or failure in screening for a trial; denial of innovative drug coverage by provincial healthcare funders; barriers associated with travel to a clinical trial (i.e., costs of traveling or relocating, inability to travel due to the COVID‐19 pandemic); medical condition of the patient (i.e., patient was considered too unwell, had a condition preventing them from being enrolled or from being able to travel to another center); trial ineligibility due to age (i.e., <12 months of age or <18 years old); drug formulation.

### 3.3 Process of application, and approvals timeframe of innovative drug access requests

Overall request approval rate was 94%, with median time from initial application to approval of 4 days (IQR25‐75 = 1–16). While 61% of requests were approved within 7 days, 14% of requests took over 4 weeks to be approved. Delay in therapy was reported in 37 requests and resulted in a change of treatment plan for 18 patients. Bridging therapy was used in 35 situations, although some of these were not due to a delay in accessing the innovative drug.

### 3.4 Funding routes

Compassionate access, through 17 different pharmaceutical companies, was obtained for 52% of requests while 27% of the requests were accessed using provincial public insurance, 13% hospital, personal or philanthropic funding, and 5% private insurance. Mechanisms of funding greatly varied between provinces with some relying mainly on compassionate access and others gaining access to provincial health insurance funding (Figure 1C). For example, in Ontario, 87% (122/141) were fulfilled using compassionate access. Provincial funding could be available as a general benefit or through specific innovative therapies mechanisms, or applications administered by individual provincial health authorities. Different funding sources were sometimes used in a sequential manner (i.e., private insurance until funding was provided by a patient support program, compassionate access until private insurance began funding, hospital or direct patient funding to allow initiation of treatment while a request was reviewed by private insurance).

## 4 DISCUSSION

Our study reports significant numbers of “out‐of‐trial” innovative therapy use for Canadian children and adolescents with cancer and provides insight on molecular targets and the therapies targeting them, as well as, barriers to access of clinical trials, and funding of innovative therapies.

### 4.1 Increasing number of innovative therapies used in pediatric oncology

Innovative therapies were widely and increasingly used “out of trial” over the past years. This was previously observed in a study of off‐label innovative therapies use for pediatric oncology in the United States over the 2007–2017^13^ period. This is consistent with adult oncology practice. It has been demonstrated that off‐label prescriptions of anticancer agents, unsupported by treatment guidelines, ranged from 7%–31% in developed countries, and patients who had exhausted standard lines of therapy were most likely to receive these prescriptions.^18^ Similarly, in the United States, 11% of patients undergoing tumor profiling under the Veterans Affairs program received off‐label prescriptions for targeted therapies.^19^ The increase in pediatric precision oncology^14^ is illustrated by the 48.3% of requests reported in this work that were based on the presence of a specific molecular driver or molecular target.

The RAS/MAP kinase pathway is the most frequently targeted pathway in our study (28% of requests). This is consistent with the findings of a recent observational study of off‐label and compassionate use in France.^20^ This aligns with the high proportion of RAS/MAP kinase pathway activation in some pediatric tumor types (i.e., low‐grade gliomas, NF‐1‐related plexiform neurofibromas, LCH) and the growing body of evidence about safety and efficacy of these compounds,^7^, ^21^ but highlights the delays in obtaining regulatory approvals and reimbursement for drugs which are already considered as standard of care by many pediatric oncologists.

Our study did not capture scientific rationale/evidence to support drug request or clinical outcomes nor did it capture the specifics of access and reimbursement for every individual situation. It is unclear whether drugs were accessed at a time when they were in early development with no published evidence or at a later stage when evidence was available but access or reimbursement was lacking. Insufficient access to clinical trials only partly explains “out‐of‐trial” access, with some agents being already proven as effective but not yet routinely available. Trials and registries of precision oncology platforms reported extended overall survival of children and adolescents benefiting from tumor profiling when treated with drugs targeting high‐priority molecular alterations.^22^, ^23^ Further collaborative work should be planned to document whether patients benefit from using these innovative therapies “out of trial” generating real‐world evidence of efficacy, safety and ideally patient‐reported outcomes including impact on quality of life.

### 4.2 Barriers to accessing clinical trials

We identified several barriers to access existing clinical trials for pediatric oncology patients, for example, patient age not meeting eligibility criteria, drug formulation, and inability to travel to study site. Awareness of these barriers is increasing, and initiatives are being designed to address them focusing on such things as age inclusivity^24^, ^25^ or implementation of decentralized trials.^26^ Here, we are providing evidence that there is limited access to clinical trials for patients with pediatric malignancies in Canada leading to other ways of access. Further efforts are needed to derive scientific knowledge from real‐world evidence. Further research focusing on specific barriers to trial enrollment is also needed.

### 4.3 Different mechanisms, processes, navigation and expertise, role of a drug access navigator

Accessing innovative therapies can be a complex and time‐consuming process. Compassionate access requests were initiated through 17 different pharmaceutical companies with different access policies, application and review processes, and contractual arrangements. Navigating this complex landscape and completing the requirements in a timely manner are crucial skills that need to be developed and secured in pediatric oncology centers. As many of these requests are for rare diseases and rare patients, it is impractical for any one physician or any one center to be knowledgeable about accessing a single innovative drug without some help or expertise.

Our study reports seemingly rapid access to innovative therapies with infrequent delays in therapy. However, bridging therapies were used in 10% of patients. The need for a support system for clinicians when seeking access to innovative therapies was previously identified in a clinician survey in the United States.^27^ This increasing complexity speaks to the importance of the drug access navigators' roles as experts in pathways to making drugs available for patients.

### 4.4 Limitations

This study is limited by its retrospective design and the capacity for centers to retrieve data about their drug access requests. Not all pediatric oncology centers in Canada were able to participate. Our intent was to report on our experience in accessing innovative therapies out of trial. Possible selection biases, recall bias (as each site was allowed to determine its own process for identifying innovative drug access requests), and missing data may preclude the validity of our sample and impact our conclusions. The proportion of approved requests (94%) may be overestimated, since unsuccessful attempts to access innovative therapies were less likely to be documented. The number of requests in earlier years (2013–2018) may be underestimated due to challenges in retrospective collection of access request data due to many factors including implementation of electronic medical records in recent years. Many pediatric oncology centers in Canada have limited involvement in the treatment of young adults, therefore, the data for patients aged 18–29 are limited. While a conservative definition of AYA in oncology would be restricted to 15–24 because of the epidemiology of pediatric cancer types, we decided to include patients up to 29 years old in order to study patients treated by pediatric centers beyond teenage years, and likely to have experienced multiple relapses or a chronic course of disease. We had fewer reports in the 18–29 category (7% of all reports) which raises concern for a selection bias. This prevents us from drawing specific conclusions from our study regarding this subset of the population. We did not capture how many requests were for an individual patient, so we do not report the number of patients included.

### 4.5 Funding and equity

We identified a variety of drug funding mechanisms with frequent use of compassionate access provided by pharmaceutical companies. Predominant access through compassionate route in major pediatric oncology centers illustrates this effective collaboration between academic centers and pharmaceutical companies. However, in the context of insufficient access to clinical trials or approved drugs, this also raises concerns in terms of equity in access to innovative therapies.

We observed that pediatric oncologists have adapted to the regulatory landscape and drug availability in order to provide the best possible treatment options to their patients. Translational expertise and collaborations were developed and there were numerous successes in accessing innovative therapies. This new field of practice leads to concerns regarding generation of scientific knowledge and equity in access. We believe that all efforts should be made to improve availability and inclusivity of clinical trials in order to address unmet needs of patients and gaps in scientific knowledge. We also acknowledge that collection of real‐world data can leverage important findings. Future research should implement a prospective methodology to measure disease response and patient reported outcomes on innovative therapies.

## AUTHOR CONTRIBUTIONS

**Sandra Judd:** Conceptualization (equal); data curation (equal); formal analysis (equal); investigation (equal); methodology (equal); project administration (equal); supervision (equal); writing – original draft (equal); writing – review and editing (equal). **Gabriel Revon‐Riviere:** Conceptualization (equal); data curation (equal); formal analysis (equal); investigation (equal); methodology (equal); project administration (equal); writing – original draft (equal); writing – review and editing (equal). **Stephanie A. Grover:** Conceptualization (equal); data curation (equal); formal analysis (equal); investigation (equal); methodology (equal); writing – original draft (equal); writing – review and editing (equal). **Rebecca J. Deyell:** Investigation (equal); methodology (equal); writing – original draft (equal); writing – review and editing (equal). **Magimairajan Issai Vanan:** Investigation (equal); writing – original draft (equal); writing – review and editing (equal). **Victor A. Lewis:** Investigation (equal); writing – original draft (equal); writing – review and editing (equal). **Lucie Pecheux:** Investigation (equal); writing – original draft (equal); writing – review and editing (equal). **Alexandra P. Zorzi:** Investigation (equal); writing – original draft (equal); writing – review and editing (equal). **Catherine Goudie:** Investigation (equal); writing – original draft (equal); writing – review and editing (equal). **Raoul Santiago:** Investigation (equal); writing – original draft (equal); writing – review and editing (equal). **Thai Hoa Tran:** Investigation (equal); writing – original draft (equal); writing – review and editing (equal). **Lesleigh S. Abbott:** Investigation (equal); writing – original draft (equal); writing – review and editing (equal). **Josee Brossard:** Investigation (equal); writing – original draft (equal); writing – review and editing (equal). **Paul Moorehead:** Investigation (equal); writing – original draft (equal); writing – review and editing (equal). **Saima Alvi:** Investigation (equal); writing – original draft (equal); writing – review and editing (equal). **Carol Portwine:** Investigation (equal); writing – original draft (equal); writing – review and editing (equal). **Avram Denburg:** Writing – original draft (equal); writing – review and editing (equal). **James A. Whitlock:** Conceptualization (equal); writing – original draft (equal); writing – review and editing (equal). **Sarah Cohen‐Gogo:** Conceptualization (equal); data curation (equal); formal analysis (equal); investigation (equal); methodology (equal); writing – original draft (equal); writing – review and editing (equal). **Daniel A. Morgenstern:** Conceptualization (equal); data curation (equal); formal analysis (equal); funding acquisition (equal); investigation (equal); methodology (equal); project administration (equal); supervision (equal); writing – original draft (equal); writing – review and editing (equal).

## FUNDING INFORMATION

Sandra Judd in the role of National Pediatric Oncology Drug Access Navigator has been supported by the PRecision Oncology For Young peopLE (PROFYLE) Program with funding from the Terry Fox Research Institute, Team Finn Foundation, and the Garron Family Cancer Centre. Gabriel Revon‐Riviere has been supported by the Atrium/CMCC Hold‘em for Life Oncology Fellowship. James A Whitlock is supported in part by the Women‘s Auxiliary Millennium Chair in Hematology/Oncology at the Hospital for Sick Children. This research did not receive any specific grant from funding agencies in the public, commercial, or not‐for‐profit sectors.

## CONFLICT OF INTEREST STATEMENT

JW: Research funding (institutional): Novartis, Daiichi Sankyo; Honoraria: Jazz, Servier. DM: Consultancy for ymAbs Therapeutics, Clarity Pharmaceuticals, RayzeBio, Regeneron and Oncoheroes Biosciences. Travel expenses Abbvie. Speaker fees from Takeda Israel Ltd. All other authors (SJ, GRR, RJD, MIV, VAL, LP, APZ, CG, RS, THT, LSA, JB, PM, SA, CP, SAG, AD, SCG) have no conflicts of interest to declare.

## Data Availability

The data that support the findings of this study are available on request from the corresponding author. The data are not publicly available due to privacy or ethical restrictions.
